# Identification of potential immunotherapy biomarkers for breast cancer by bioinformatics analysis

**DOI:** 10.1042/BSR20212035

**Published:** 2022-02-04

**Authors:** Yao Song, Meiling Lu, Lijin Feng, Qian Chen, Hua Huang, Qing Lin

**Affiliations:** 1 Department of Radiation Oncology, Tenth People’s Hospital Affliated to Tongji University, Shanghai 200072, China; 2 Department of Central Laboratory, Shanghai Tenth People’s Hospital of Tongji University, Shanghai 200072, China; 3 Department of Pathology, Tenth People’s Hospital Affiliated to Tongji University, Shanghai 200072, China; 4 Department of Pathology, Affiliated Hospital of Nantong University, Nantong 226300, Jiangsu Province, China

**Keywords:** bioinformatics analysis, biomarkers, Breast cancer, immunotherapy, prognosis

## Abstract

Breast cancer is a serious malignancy with a high incidence worldwide and a tendency to relapse. We used integrated bioinformatics analysis to identify potential biomarkers in breast carcinoma in the present study. Microarray data, 127breast tumor samples and 23 non-tumor samples, received from the Gene Expression Omnibus (GEO) dataset; 121 differentially expressed genes (DEGs) were selected. Functional analysis using DAVID revealed that these DEGs were highly gathered in endodermal cell differentiation and proteinaceous extracellular matrix. Five bioactive compounds (prostaglandin J2, tanespimycin, semustine, 5182598, and flunarizine) were identified using Connectivity Map. We used Cytoscape software and STRING dataset to structure a protein–protein interaction (PPI) network. The expression of CD24, MMP1, SDC1, and SPP1 was much higher in breast carcinoma tissue than in Para cancerous tissues analyzed by Gene Expression Profiling Interactive Analysis (GEPIA) and ONCOMINE. Overexpression ofCD24, MMP1, SDC1, and SPP1 indicated the poor prognosis in breast carcinoma patients analyzed by Kaplan–Meier (KM) Plotter. Immunohistochemistry microarray was used to further confirm that protein expression of CD24, MMP1, SDC1, and SPP1 was much higher in tumor sections than in Para cancerous tissues. Hub genes expression at the protein level was correlated tothe breast cancer subtype and grade. Furthermore, immunity analysis showed that CD24, MMP1, SDC1, and SPP1 were potentially associated with five immune cell types infiltration (CD8+ T cells, CD4+ T cells, neutrophils, macrophages,and dendritic cells) by TIMER. Thus, this study indicates potential biomarkers that could have applications in the development of immune therapy for breast cancer. However, further studies are required for verifying these results *in vivo* and *vitro*.

## Introduction

Breast cancer occurs frequently in non-skin carcinoma and leading to death in America females at the second level, with 268600 new patients and 41760 deaths in 2019, accounting for up to 30% of all new cancers and 15% of cancer deaths [1]. Risk factors for breast cancer include age, environment, smoking, and inheritance; approximately 5–10% of cases are due to genes inherited from the patient’s parents, such as BRAC1 and BRAC2 mutations [2,3]. There are four subtypes of breast cancer based on molecular characteristics: luminal A, luminal B, triple negative and HER2 overexpressing. Besides the traditional treatment methods of surgery, chemotherapy and/or radiation therapy used for breast cancer, endotherapy can be used in HR+ cases. Anti-HER-2 therapy is commonly used in HER2+ patients, and in the past several years, immunotherapy has been commonly used to treat triple-negative breast cancer [4].

Although the survival rate has improved quickly with the development of surgical techniques and exploration of new targeted drugs, the effects of therapies for advanced breast cancer have remained poor, only 27% for a 5-year survival rate [5]. Hence, investigation the mechanism of cancer progression and finding the potential prognostic biomarkers is very important.

High-throughput microarray technology and bioinformatics analysis can be used to identify differences in gene expression between cancerous and para- cancerous tissues, analyze the DEGs, and identify the pathways leading to tumorigenesis and cancer progression.

To understand the molecular mechanisms associated with breast cancer progression, we performed bioinformatics analysis using the GEO and The Cancer Genome Atlas (TCGA) databases to review all DEGs in breast cancer, for identifying prognostic biomarkers and potential molecular targets. Then, tissue microarray analysis (TMA) was used to validate the protein expression of hub genes. Our results indicated that MMP1, CD24, SDC1, and SPP1 are potential novel prognostic biomarkers and candidate immunotherapy targets for breast cancer.

## Materials and methods

### Breast cancer data preparation

Microarray platform (GLP570)[6] was used to collect two independent breast cancer gene expression profiles, GSE26910 [7] and GSE42568 [8], which contain 127 breast carcinoma samples and 23 non-cancer samples. These data were analyzed using the Affymetrix Human Genome U133 Plus 2.0 Array [transcript (gene) version; Santa Clara, CA, U.S.A.]. Furthermore, we used 1,105 samples in breast carcinoma and 113 samples in para-cancerous from TCGA dataset for validation.

### Identification of DEGs

After downloading the datasets from GEO, the GEO2R online tool was used to find the DEGs between breast cancer tissue and non-tumor tissue. The conditions for screening of the DEGs were *P*<0.05 and |log (fold change)| > 2. An online Venn diagram tool was used to identify up- and down-regulated genes in the two datasets.

### Identification of potential drugs

CMap was used to identify possible drugs that could be used to inhibit tumor progression in breast cancer patients based on targeting the discovered DEGs [9]. First, we arranged the lists of up- and down-regulated genes for both tumor and non-tumor tissues and uploaded the genes to the CMap online tool. Next, an enrichment score, representing the similarity in up- and down-regulated genes, were calculated for each drug. A positive enrichment score indicates that the drug can exacerbate breast cancer progression, whereas a negative enrichment number means that the drug may inhibit tumor formation.

### Functional enrichment analysis

Gene ontology (GO) terms and Kyoto Encyclopedia of Genes and Genomes (KEGG) pathways correlated to the DEGs were identified by DAVID online tool [10]. GO analysis describes genes in terms of the related molecular functions, biological processes, and cellular components [11]. KEGG pathway analysis was used to check their reference pathways in the indicated genes [12].

### PPI network construction

STRING was used for construction a PPI network and molecular function network for the DEGs [13]. After collecting data for the PPI network, visualized the network by Cytoscape, distinguished whether the highly connected modules were molecular complexes or clusters by the plugin Molecular Complex Detection (MCODE).

### Selection of hub genes

We checked the 32 selected genes expression in tumor tissues and non-tumor tissues based on TCGA data by GEPIA [14]. The DEGs with significantly differential expression between the two tissue types were selected for further analysis. KM Plotter can analysis the effect of 54675 genes on patient survival in 18674 cancer samples, mainly based on the GEO, TCGA, and EGA databases [15]. The associations of the DEGs verified by GEPIA with survival rates of breast cancer patients were explored by KM Plotter; the DEGs for which higher expression indicated significantly worse survival were selected as hub genes. *P*<0.05 looked as statistical significance. ONCOMINE dataset, confirmed the focus genes expression between cancer and para cancerous tissues in breast cancer [16].

### Gene set enrichment analysis (GSEA)

We separated breast carcinoma tissues in two groups through the hub genes median expression on TCGA dataset. GSEA[17] was used to identify potential functions for hub genes. KEGG pathways associated with the up- and down-regulated genes were selected. The cut-off condition was considered for adjusted P<0.05.

### Associations of immune cell infiltration with hub gene expression

TIMER used for systematically analyzing the relation between immune infiltration and hub gene expression [18]. Hub gene expression, the correlations with the infiltration of immune cells were evaluated by “Gene module”.

### Immunohistochemistry validation

A total of 657 breast carcinoma samples and paired para-cancerous samples were collected from 2013 to 2018 at Tenth Hospital of Tongji University, China. Pathologic diagnoses and classifications were made according to the UICC Classification of Malignant Tumors.

Tissue cores were obtained from the formalin-fixed paraffin-embedded (FFPE) blocks; the diagnosis of breast tumor was based on review by a pathologist after coloration with Hematoxylin and Eosin. TMA analysis (Shanghai Outdo Biotech, Shanghai, China) checked by 2-mm tissue cores two areas of the tumor in each patients(invasive margin and tumor bulk). Ethical approval for this study was obtained from The Human Research Ethics Committee of Tenth Hospital of Tongji University. All patients provided informed consent.

Antigen retrieval was conducted by microwave pre-treatment in EDTA buffer (pH 9.0) for 20 min; then, endogenous peroxidase was removed using 3% H_2_O_2_, after that blocking for 20 min with avidin. Furthermore, rabbit polyclonal antibodies against MMP1 (ab52631, Abcam, UK; dilution 1:60) incubated on the slides overnight at 4 °C, CD24 (ab31622, Abcam, U.K.; dilution 1:100), SDC1 (ab7280, Abcam, U.K.; dilution 1:500), and SPP1 (ab214050, Abcam, U.K.; dilution 1:500) for 24 h, before being incubated with secondary antibodies at room temperature for 30 min. Tissue sections were incubated with 3,3′-diaminobenzidine for 10 min, then counterstained, dehydrated, and mounted.

Reading slides by a Nano-Zoomer 2.0-HT slide scanner (Hamamatsu Photonics K.K., Hamamatsu, Japan), analyzing the images by the NDP. Captured images in TMA slides. The tissue immunostaining was read independently by two trained pathologists. The criteria for immunohistochemistry evaluation were as follows. 0, negative; 1, weak; 2, medium; and 3, strong scored as the staining intensity; 0, 0%; 1, 1–25%; 2, 26–50%; 3, 51–75%; and 4, 76–100% scored as the staining extent, which counted by the percentage of positively stained areas in relation to the whole cancer area. Sum of the extent score and the intensity score as the final expressed results, which was graded as follows: −, score 0–2; +, score 3 or 4; ++, score 5 or 6; or +++, score 7. Here, − and 1+ represent low expression; and 2+ and 3+ represent high expression.

## Results

### DEGs in breast cancer

**Figure 1:**
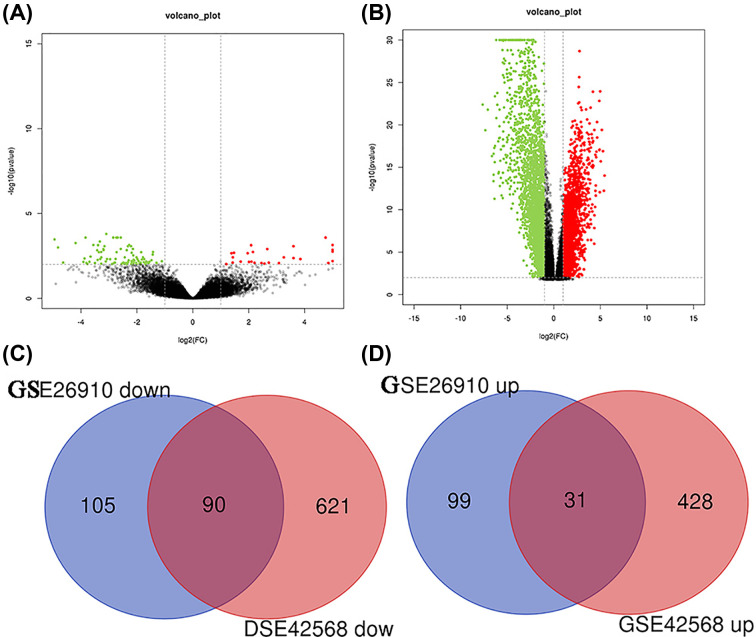
DEGs in breast cancer and non-cancer tissues (**A**) Volcano plot, represented DEGs in breast cancer tissues and non-tumor samples in GSE26910 dataset. (**B**)Volcano plot, represented DEGs in breast cancer tissues and non-tumor tissues in GSE42568 dataset. Red dots, indicate genes highly induced in breast cancer; green dots indicate genes greatly reduced in breast cancer, blue dots indicate non-DEGs. (**C**)Venn diagram represented the downregulated overlapping DEGs from GSE26910 and GSE42568 datasets. (**D**) Venn diagram represented the upregulated overlapping DEGs from GSE26910 and GSE42568 datasets.

GSE26910 and GSE42568 datasets were obtained in the GEO database, containing 127 breast carcinoma tissues and 23 non-tumor samples. There were 325 DEGs (130 up-regulated and 195 down-regulated) obtained from GSE26910, and 1170 DEGs (459 up-regulated and 711 down-regulated) from GSE42568 between tumor and non-tumor tissues. These results are shown as volcano plots in Figure 1A,B. Furthermore, the DEGs were analyzed by Venn diagram; 121 DEGs (31 up-regulated and 90 down-regulated) were found in the two datasets, as shown in Figure 1C,D and Supplementary Table S1.

### DEGs enrichment analysis

DAVID online was used to further analyze the functions of the overlapping two GEO datasets DEGs in breast cancer tissues. The up-regulated DEGs were mainly enriched in endodermal cell differentiation, collagen catabolic process, and cell adhesion (biological processes); proteinaceous extracellular matrix (ECM; cellular component); and lipid binding (molecular function). Down-regulated DEGs were mainly enriched in negative regulation of cell proliferation, extracellular region, and lipid binding. In the KEGG pathway analysis, DEGs were mainly enriched in ECM–receptor interaction and TNF signaling pathway (Tables 1 and 2).

**Table 1:** GO analysis of DEGs in breast cancer

Expression	Category	Term	Count	%	*P*-value	FDR
**Up-regulated**	GOTERM_BP_DIRECT	GO:0035987∼endodermal cell differentiation	4	9.15	1.32E-05	0.017315
	GOTERM_BP_DIRECT	GO:0030574∼collagen catabolic process	3	5.86	2.05E-04	0.268194
	GOTERM_BP_DIRECT	GO:0007155∼cell adhesion	5	1.14	2.44E-04	0.319249
	GOTERM_BP_DIRECT	GO:0002063∼chondrocyte development	3	6.86	2.82E-04	0.368791
	GOTERM_BP_DIRECT	GO:0001502∼cartilage condensation	3	6.86	4.20E-04	0.548872
	GOTERM_CC_DIRECT	GO:0005578∼proteinaceous extracellular matrix	8	18.30	1.35E-08	1.30E-05
	GOTERM_MF_DIRECT	GO:0008289∼lipid binding	6	4.84	4.06E-04	0.516306
	GOTERM_MF_DIRECT	GO:0001077∼transcriptional activator activity, RNA polymerase II core promoter proximal region sequence-specific binding	7	5.65	4.35E-04	0.056502
**Down- regulation**	GOTERM_BP_DIRECT	GO:0008285∼negative regulation of cell proliferation	10	8.07	6.54E-05	0.101387
	GOTERM_BP_DIRECT	GO:0006869∼lipid transport	5	4.04	3.69E-04	0.570794
	GOTERM_BP_DIRECT	GO:0050873∼brown fat cell differentiation	4	3.23	3.87E-04	0.597749
	GOTERM_BP_DIRECT	GO:0045429∼positive regulation of nitric oxide biosynthetic process	4	3.23	9.29E-04	1.430151
	GOTERM_CC_DIRECT	GO:0005576∼extracellular region	21	1.70	1.50E-05	0.016859
	GOTERM_CC_DIRECT	GO:0005615∼extracellular space	19	1.54	1.67E-05	0.018833
	GOTERM_MF_DIRECT	GO:0008289∼lipid binding	6	4.84	4.06E-04	0.516306
	GOTERM_MF_DIRECT	GO:0001077∼transcriptional activator activity, RNA polymerase II core promoter proximal region sequence-specific binding	7	5.65	4.35E-04	0.553146

Abbreviations: BP, biological process; CC, cell component; MF, molecular function.

**Table 2:** KEGG pathway analysis of DEGs in breast cancer

Pathway ID	Category	Count	%	*P*-value	Genes
bta04512	ECM–receptor interaction	7	4.18	1.85E-05	*SDC1, CD36, COMP, ITGA7, COL11A1, FN1, SPP1*
bta04668	TNF signaling pathway	6	3.60	6.31E-04	*FOS, PTGS2, MMP9, CXCL2, CXCL10*
bta05200	Pathways in cancer	9	5.37	0.003734	*EGFR, FOS, EDNRB, BMP2, PTGS2, MMP9, ZBTB16, MMP1, FN1*
bta05144	Malaria	4	2.39	0.004793	*SDC1, CD36, COMP, HBB*
bta03320	PPAR signaling pathway	4	2.39	0.01037	*CD36, FABP4, ADIPOQ, MMP1*
bta05205	Proteoglycans in cancer	6	3.58	0.009768	*EGFR, SDC1, GPC3, ERBB3, MMP9, FN1*
bta04510	Focal adhesion	6	3.58	0.010782	*EGFR, COMP, ITGA7, COL11A1, FN1, SPP1*

### Identification of small molecular drugs

CMap was used to identify small molecular drugs according to the up- and down-regulated DEGs. Prostaglandin J2, tanespimycin, semustine, 5182598, and flunarizine were the five small molecules most significantly negatively correlated with the DEGs in breast cancer. Thus, these molecules are potentially targeted drugs for breast cancer (Table 3).

**Table 3:** CMap results

Rank	CMap name	Mean	*n*	Enrichment	*P*-value
1	Quinpirole	0.717	15	0.945	0
2	15-δ prostaglandin J2	−0.442	62	−0.583	0
3	Tanespimycin	−0.294	4	−0.378	0
4	Semustine	−0.637	2	−0.903	0.00016
5	5182598	−0.713	4	−0.988	0.00036
6	Flunarizine	−0.624	4	−0.882	0.00048
7	Propafenone	−0.588	4	−0.863	0.00064
8	Securinine	−0.641	4	−0.842	0.00109
9	Altizide	0.45	2	0.83	0.00127
10	5224221	−0.7	4	−0.975	0.00135
11	Scopolamine	0.382	6	0.824	0.00151
12	Lanatoside C	−0.542	4	−0.696	0.00193
13	Pridinol	−0.54	4	−0.816	0.00209
14	Mesoridazine	−0.53	3	−0.816	0.00209
15	Sulfaquinoxaline	0.589	15	0.888	0.00264

### PPI network and module analysis

**Figure 2:**
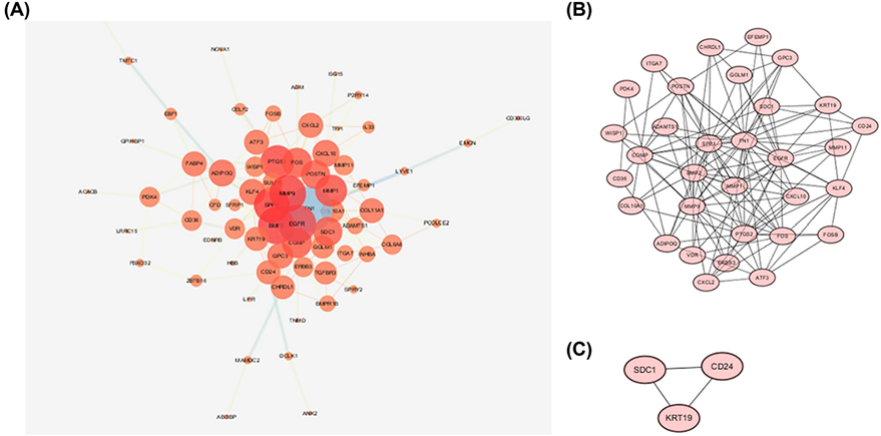
PPI network and module analysis (**A**) PPI network. (**B**) Top module cluster analyzed by MCODE. (**C**) Top two module clusters analyzed by MCODE.

We identified protein connections between the overlapping DEGs in STRING online database; connection score was 0.2. Next, Cystoscope was used to establish a PPI network consisting of 32 nodes and 134 edges (Figure 2A). MCODE was then used to find two clusters: cluster 1 contained 32 nodes and 134 edges, with a score of 8.645; cluster 2 contained 3 nodes with 3 edges, with a score of 3 (Figure 2B,C).

### Hub genes survival analysis

**Figure 3:**
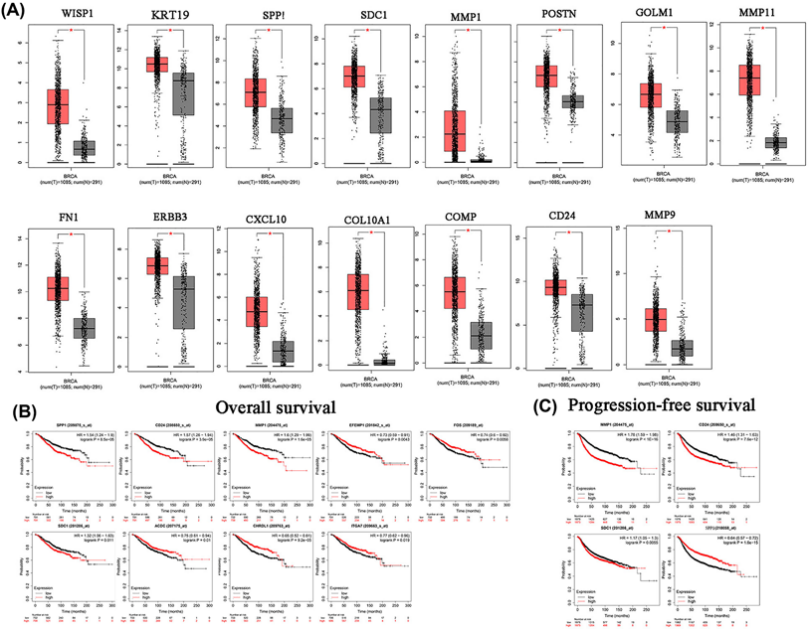
Survival analysis for hub genes (**A**) 15 hub genes expressed much higher in breast cancer tissues than normal breast tissues by GEPIA. (**B**)9 hub genes was correlated with OS in breast cancer patients. (**C**) MMP1, CD24, SDC1, and SPP1 was expression correlated with RFS in breast cancer patients. Abbreviation: OS, overall survival; RFS, relapse-free survival. *,*P*<0.01.

GEPIA online tool was used for checking the hub genes expression in breast cancer and non-tumor tissues by TCGA dataset. CD24, MMP1, MMP9, SDC1, COMP, GOLM1, POSTN, FN1, SPP1, CXCL10, WISP1, ERBB3, COL10A1, KRT19 and MMP11 were upregulated in breast cancer tissues compared with normal tissues (Figure 3A). FOSB, FOS, PTGS2, EGFR, EFEMP1, KLF4, CXCL2, GPC3, ITGA7, CHRDL1, CD36, PDK4, BMP2, ATF3, ADIPOQ, and ADAMTS1 expression was much lower in tumor tissues than in normal tissues (Supplementary Figure S1).

KM Plotter was used to check the prognostic value of the 33 DEGs. Higher MMP1, SDC1, SPP1, and CD24 expression was correlated with worse overall survival (OS) and relapse-free survival (RFS) in breast cancer patients (Figure 3B,C). Other genes showed no significant correlation with survival (Supplementary Figure S2). Hence, we focused on these four genes in the subsequent analysis.

**Figure 4:**
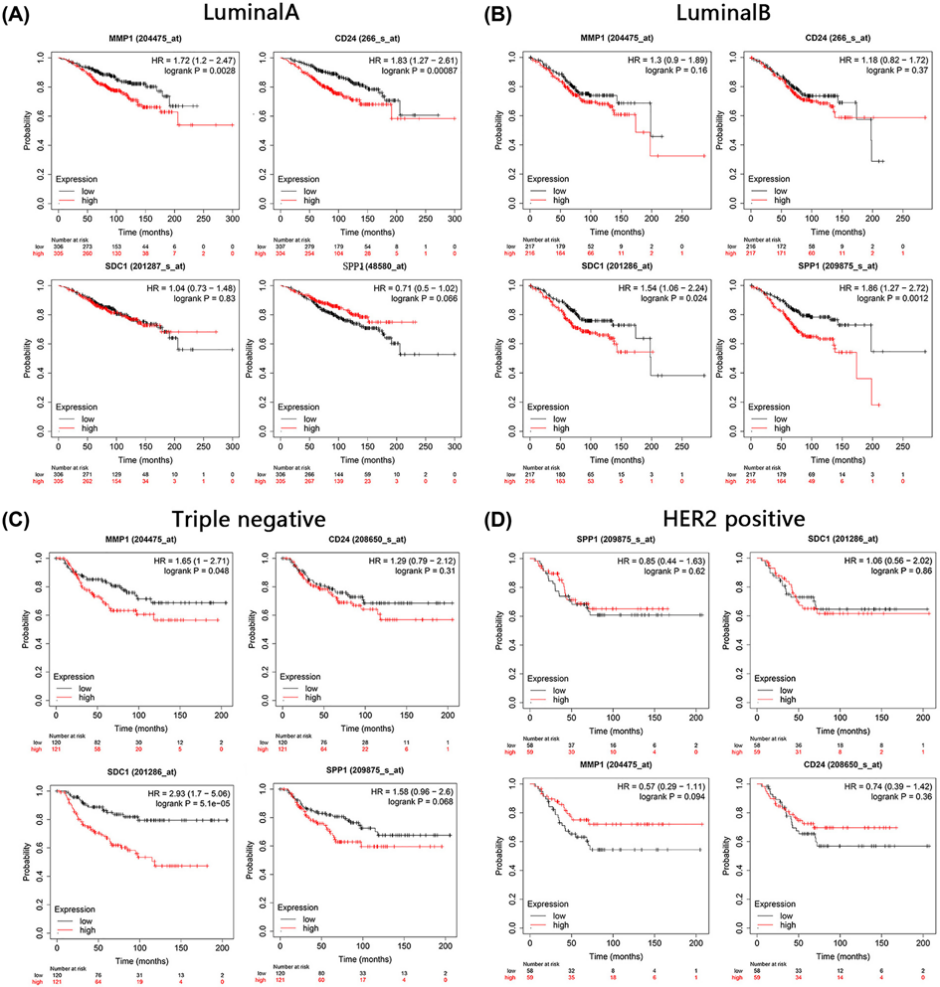
OS curves for MMP1, CD24, SDC1, and SPP1 expression in breast cancer subtypes (**A**) Luminal A; (**B**) luminal B; (**C**) triple-negative; (**D**) HER2+.

In the luminal A subtype, higher MMP1 and CD24 expression levels were correlated with worse OS. In the luminal B subtype, SDC1 and SPP1 expression was significantly correlated with prognosis of breast cancer patients. MMP1 and SDC1 expression was correlated with prognosis in the triple-negative subtype. In HER-2+ breast cancer, MMP1, SDC1, SPP1, and CD24 expression showed no significant association with prognosis (Figure 4A–D). None of the four genes had any significant correlation with tumor stages I–IV, as shown in Supplementary Figure S3.

### GSEA

**Figure 5:**
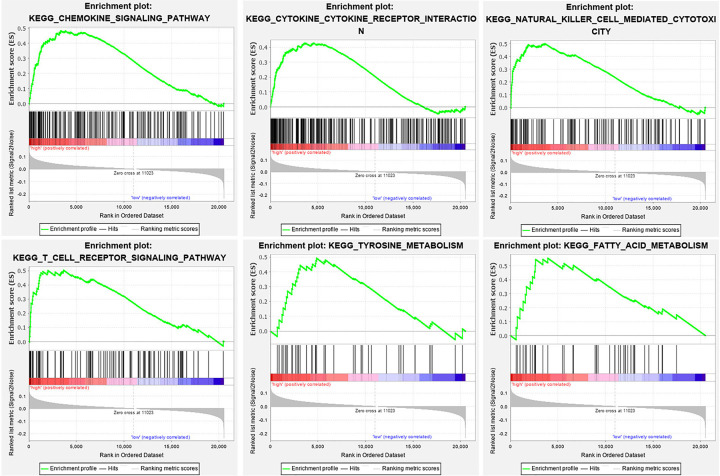
GSEA was applied to identify enriched biological processes for the four key genes (MMP1, CD24, SDC1 and SPP1) with highly expressed samples

To find the potential function of the four genes according to the TCGA breast cancer dataset, we evaluated the KEGG pathways involved in the highly expressed samples by GSEA. The most enriched KEGG pathways were chemokine signaling pathway, cytokine receptor interaction, natural killer cell-mediated cytotoxicity, and T cell receptor signaling pathway (Figure 5).

### Immune infiltration and hub genes

**Figure 6:**
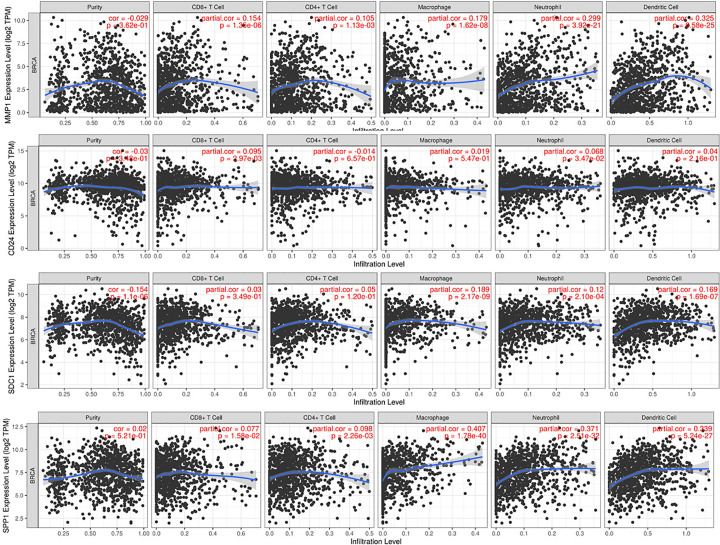
Correlations of expression of four key genes (*MMP1*, *CD24*, *SDC1*, and *SPP1*) with immune cell infiltration

Immune cell infiltration was analyzed with respect to its associations with MMP1, SDC1, SPP1, and CD24 expression. MMP1 expression was positively associated with infiltration of CD4+ T cells (rho = 0.105, *P*=1.13e-03), CD8+ T cells (rho = 0.154, *P*=1.35e-05), macrophages (rho = 0.179, *P*=1.62e-8), neutrophils (rho = 0.299, *P*=3.92e-21), and dendritic cells (DCs; rho = 0.325, *P*=6.58e-25). CD24 expression was positively correlated with infiltration of CD8+ T cells (rho = 0.095, *P*=2.97e-03) and neutrophils (rho = 0.068, *P*=3.47e-02). SDC1 expression was significantly positive associated with CD8+ T cells (rho = 0.171, *P*=6.16e-08), macrophages (rho = 0.189, *P*=2.17e-09), neutrophils (rho = 0.12, *P*=2.1e-4), and DCs (rho = 0.169, *P*=1.69e-07). SPP1 expression higher, the infiltration of CD4+ T cells (rho = 0.098, *P*=2.26e-03), CD8+ T cells (rho = 0.077, *P*=1.58e-02), macrophages (rho = 0.407, *P*=1.78e-40), DC (rho = 0.339, *P*=5.24e-27), and neutrophils (rho = 0.371, *P*=2.51e-32) was much more (Figure 6).

TIMER survival model can be used for analyzing the clinical relevance of one or more tumor immune subsets. In the multivariable Cox proportional hazard model, in BRCA, stage 3 (*P*=0.000), stage 4 (*P*=0.000), CD24 (*P*=0.002), and MMP1 (*P*=0.042) were significantly correlated with survival. Then, we performed subtype analysis; the BRCA-basal group comprised 118 patients with the following associations with survival: stage (*P*=0.000), purity (*P*=0.000), CD4+ T cells infiltration (*P*=0.000), DC infiltration (*P*=0.001), SDC1 expression (*P*=0.025), and SPP1 expression (*P*=0.037). In the BRCA-luminal group, 591 patients were analyzed; stage (*P*=0.000), DC infiltration (*P*=0.008), SDC1 expression (*P*=0.014), CD24 expression (*P*=0.028), and MMP1 expression (*P*=0.001) were correlated with survival. However, in the BRAC-HER2 group, no clinically relevant associations with survival were found among the 65 patients (Tables 4-7).

**Table 4:** The Cox proportional hazard model of SDC1, SPP1, CD24, MMP1 and tumor-infiltrating immune cells in breast carcinoma (TIMER)

BRCA	Coef	HR	95% CI-l	95% CI-u	*P*-value
Stage 2	0.355	1.426	0.757	2.688	0.272
Stage 3	1.211	3.357	1.753	6.427	0.000*
Stage 4	2.418	11.218	5.036	24.991	0.000*
Purity	0.412	1.509	0.542	4.204	0.431
CD8-T cell	−1.126	0.324	0.018	5.839	0.445
CD4-T cell	2.406	11.094	0.135	914.872	0.285
Macrophage	2.619	13.717	0.759	247.73	0.076
Neutrophil	0.986	2.68	0.008	897.384	0.74
Dendritic	−1.445	0.236	0.028	2.002	0.186
SDC1	−0.031	0.969	0.793	1.185	0.762
SPP1	−0.035	0.966	0.837	1.114	0.633
CD24	0.189	1.208	1.07	1.363	0.002*
MMP1	0.108	1.114	1.004	1.237	0.042*

^*^,*P*<0.05.

**Table 5:** The Cox proportional hazard model of SDC1, SPP1, CD24, MMP1 and tumor-infiltrating immune cells in basal like breast carcinoma (TIMER)

BRCA-basal	Coef	HR	95% CI-l	95% CI-u	*P*-value
Stage 2	9.755	1724.45	5628.5	52833.27	0.000*
Stage 3	11.601	10916.83	36549.6	3.26*10⁁5	0.000*
Stage 4	13.302	59858.1	53221.18	6.73*10⁁6	0.000*
Purity	6.405	605.04	21.91	16710.19	0.000*
CD8-T cell	−1.209	0.298	0.003	28.12	0.602
CD4-T cell	18.874	157415300	412998.97	5.999*10⁁10	0.000*
Macrophage	4.015	55.4	0.141	21703.2	0.188
Neutrophil	1.417	4.13	0.023	737.4	0.592
Dendritic	−2.981	0.051	0.008	0.311	0.001*
SDC1	0.623	1.865	1.082	3.214	0.025*
SPP1	0.352	1.522	1.022	1.98	0.037*
CD24	−0.339	0.712	0.466	1.09	0.118
MMP1	−0.137	0.872	0.794	1.095	0.237

^*^,*P*<0.05.

**Table 6:** The Cox proportional hazard model of SDC1, SPP1, CD24, MMP1 and tumor-infiltrating immune cells in luminal breast carcinoma (TIMER)

BRCA-luminal	Coef	HR	95% CI-l	95% CI-u	*P*-value
Stage 2	0.042	1.043	0.503	2.161	0.91
Stage 3	0.745	2.107	0.945	4.7	0.069
Stage 4	2.15	8.583	3.164	23.29	0.000*
Purity	−0.384	0.681	0.164	2.835	0.598
CD8-T cell	1.523	4.587	0.047	451.93	0.515
CD4-T cell	2.937	18.865	0.011	31150.4	0.437
Macrophage	2.201	9.034	0.155	526.65	0.289
Neutrophil	8.566	5248.648	0.236	1.17*10⁁8	0.093
Dendritic	−5.114	0.006	0.000	0.256	0.008*
SDC1	−0.343	0.71	0.54	0.933	0.014*
SPP1	−0.032	0.969	0.797	1.177	0.75
CD24	0.168	1.183	1.018	1.375	0.028*
MMP1	0.284	1.132	0.794	1.56	0.001*

^*^,*P*<0.05.

**Table 7:** The Cox proportional hazard model of SDC1, SPP1, CD24, MMP1 and tumor-infiltrating immune cells in HER2+ breast carcinoma (TIMER)

BRCA-HER2	Coef	HR	95% CI-l	95% CI-u	P value
Stage 2	15.68	6.45*10⁁6	0.000	Inf	0.999
Stage 3	19.574	3.168*10⁁8	0.000	Inf	0.999
Stage 4	20.431	7.466*10⁁8	0.000	Inf	0.999
Purity	4.751	115.7	0.525	2.548*10⁁4	0.084
CD8-T cell	−2.266	0.104	0.000	8.899*10⁁7	0.829
CD4-T cell	14.213	1.487*10⁁6	0.000	3.54*10⁁18	0.328
Macrophage	4.79	120.34	0.000	1.234*10⁁13	0.711
Neutrophil	−10.241	0.000	0.000	3.602*10⁁12	0.608
Dendritic	−0.139	0.87	0.000	1.299*10⁁5	0.982
SDC1	−1.066	0.345	0.135	0.881	0.026*
SPP1	−0.377	0.686	0.301	1.565	0.371
CD24	0.521	1.684	0.761	3.726	0.198
MMP1	0.123	1.131	0.559	2.288	0.732

*,*P*<0.05.

### Validation of hub gene expression in different samples

**Figure 7:**
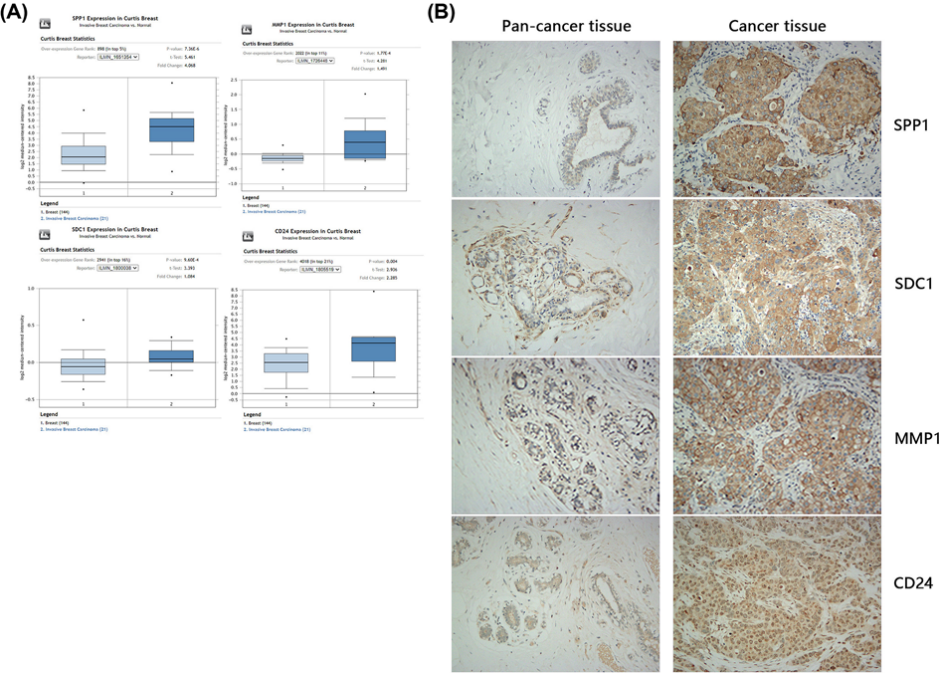
Evaluation of MMP1, CD24, SDC1, and SPP1 expression in breast cancer tissue and normal tissue (**A**) MMP1, CD24, SDC1, and SPP1 expression was up-regulated in breast cancer tissue compared with normal tissue in the Oncomine dataset. (**B**) MMP1, CD24, SDC1, and SPP1 protein expression was up-regulated in breast cancer tissue compared with normal tissue according to tissue microarray analysis.

ONCOMINE, used for confirming the selected four DEGs in breast tumor and non-tumor tissues at the mRNA level. MMP1, SDC1, SPP1, and CD24 mRNA declaration was much higher in tumor tissues than in non-tumor tissue (Figure 7).

Furthermore, a tissue microarray was used for verifying the protein levels of these four hub genes in breast cancer and para cancerous tissues. High expression of MMP1 was found in 30.7% of cancer tissues but 1.2% of pan-cancer tissues (*P*=0.00). The percentages of high protein expression of CD24, SDC1, and SPP1 were 28.6, 34.2, and 19.2% in breast cancer tissue, compared with 1.5, 1.5, and 1.1% in pan-cancer tissue, respectively (*P*=0.00, *P*=0.00, and *P*=0.00) (Table 8, Figure 5B). The protein expression of MMP1, CD24, SDC1, and SPP1 was significantly correlated with tumor grade and subtype but not with tumor stage (Tables 9-12). In the molecular correlation analysis, MMP1 protein was found to be significantly correlated with SPP1 protein expression (*P*=0.03) but not with that of SDC1 or CD24 (*P*=0.33 and *P*=0.74); CD24 protein expression was significantly correlated with SDC1 and SPP1 (*P*=0.00 and *P*=0.00) but not MMP1 protein expression (*P*=0.74); SDC1 protein expression was significantly correlated with SDC1 and SPP1 (*P*=0.00, *P*=0.00) but not MMP1 protein expression (*P*=0.33); and SPP1 protein expression was significantly correlated with MMP1, CD24, and SDC1 protein expression (*P*=0.03, *P*=0.00, and *P*=0.00) (Tables 9-12).

**Table 8:** MMP1, CD24, SDC1 and SPP1 protein expression in cancer and paracancerous tissues

	Paracancerous tissue (%)	Cancer tissue (%)	χ^2^	*P*
SPP1			117.316	0.00
Low	644 (98.9)	531 (80.8)		
High	7 (1.1)	126 (19.2)		
SDC1			237.389	0.00
Low	641 (98.5)	432 (65.8)		
High	10 (1.5)	225 (34.2)		
CD24			186.649	0.00
Low	641 (98.5)	469 (71.4)		
High	10 (1.5)	188 (28.6)		
MMP1			211.385	0.00
Low	643 (98.8)	455 (69.3)		
High	8 (1.2)	202 (30.7)		

**Table 9:** MMP1 expression in carcinoma tissue and the correlation with clinical factors

Characteristics	MMP1 (low)	MMP1 (high)	*P*-value
Age (years)			
≤50			0.12
>50	333	157	
	120	41	
Grade			**0.01** *
0/I	6	6	
II	207	113	
III	197	65	
Vascular invasion			0.76
N	378	167	
Y	73	30	
Stage			0.24
I	162	60	
II	198	93	
III	83	41	
Subtype			**0.00** **
Luminal A	194	114	
Luminal B	92	41	
HER2-positive	64	14	
Triple-negative	99	24	
N			0.66
Negative	264	111	
Positive	187	85	
CD24			0.74
Low	328	51	
High	123	145	
SDC1			0.33
Low	303	124	
High	148	72	
SPP1			**0.03** *
Low	376	149	
High	75	47	

^*^,*P*<0.05;

^**^,*P*<0.01.

**Table 10:** CD24 expression in carcinoma tissue and the correlation with clinical factors

Characteristics	CD24 (low)	CD24 (high)	*P*-value
Age (years)			0.12
≤50	351	139	
>50	122	37	
Grade			**0.00** **
0/I	11	1	
II	254	66	
III	172	90	
Vascular invasion			0.78
N	399	146	
Y	74	29	
Stage			0.36
I	167	56	
II	210	81	
III	96	35	
Subtype			**0.00** **
Luminal A	256	52	
Luminal B	102	31	
HER2-positive	38	40	
Triple-negative	77	48	
N			0.70
Negative	272	103	
Positive	201	71	
MMP1			0.74
Low	328	123	
High	145	51	
SDC1			**0.00** **
Low	330	97	
High	143	77	
SPP1			**0.00** **
Low	400	125	
High	73	49	

^**^,*P*<0.01.

**Table 11:** SDC1 expression in carcinoma tissue and the correlation with clinical factors

Characteristics	SDC1 (low)	SDC1 (high)	*P*-value
Age (years)			0.86
≤50	322	168	
>50	107	54	
Grade			0.00**
0/I	11	1	
II	228	92	
III	152	110	
Vascular invasion			0.07
N	359	186	
Y	67	36	
Stage			0.36
I	153	71	
II	187	106	
III	88	44	
Subtype			**0.00** **
Luminal A	239	71	
Luminal B	92	43	
HER2-positive	30	50	
Triple-negative	67	58	
N			0.45
Negative	253	124	
Positive	176	98	
MMP1			0.33
Low	303	148	
High	124	72	
CD24			**0.00** **
Low	330	143	
High	97	77	
SPP1			**0.00** **
Low	371	156	
High	58	66	

^**^,*P*<0.01.

**Table 12:** SPP1 expression in carcinoma tissue and the correlation with clinical factors

Characteristics	SPP1 (low)	SPP1 (high)	*P*-value
Age (years)			0.28
≤50	392	98	
>50	135	26	
Grade			0.00**
0/I	12	0	
II	277	43	
III	191	71	
Vascular invasion			0.37
N	444	101	
Y	80	23	
Stage			0.36
I	182	42	
II	236	57	
III	109	23	
Subtype			0.00**
Luminal A	273	37	
Luminal B	109	26	
HER2-positive	54	26	
Triple-negative	90	35	
N			0.29
Negative	300	77	
Positive	227	47	
MMP1			0.03*
Low	376	75	
High	149	47	
CD24			0.00**
Low	400	73	
High	125	49	
SDC1			0.00**
Low	371	58	
High	156	66	

^*^,*P*<0.05;

^**^,*P*<0.01.

## Discussion

In this study, two microarray datasets GSE26910 and GSE42568 were collected from GEO database, and a total of 121 DEGs between breast cancer and non-cancer tissues were identified, comprising 31 up-regulated and 90 down-regulated genes. Bioinformatics analysis was conducted based on these DEGs.

First, several target small molecules that could be used for inhibition of breast cancer development were identified. Prostaglandin J2, the endogenous product of the cyclooxygenase pathway, mediated pro- and anti-inflammatory effects through receptor-dependent or -independent pathways. Moreover, 15-deoxy-δ (12,14)-prostaglandin J (2), one of the main subtypes of prostaglandin J2, inhibited cancer growth through arresting cell growth in G_2_/M phase and inducing apoptosis of breast cancer cells [20]. However, this requires further exploration through *in vivo* and clinical studies. Tanespimycin, an inhibitor of heat shock protein 90, combination with trastuzumab showed higher anticancer affection in metastatic breast cancer patients for HER2+ molecular subtype, compared with trastuzumab alone in a phase II study [19]. However, tanespimycin had no effect in metastatic or locally advanced, unresectable breast cancer in a phase II study involving three patients. Hence, further study of tanespimycin in unselected breast cancer patients were not recommended [20]. However, tanespimycin is a potential targeted therapy drug in selected breast cancer populations. Semustine, a 4-methyl derivative of lomustine, is widely used in glioma therapy [21]. The growth of MCF-7 breast carcinoma cells was shown to be inhibited by semustine, but the mechanism and effects of semustine in vivo remain unclear [22]. A benzylisoquinoline alkaloid, 5182598, is considered to be an important anticancer drug because it can repair damaged metabolic pathways in metastatic prostate carcinoma [23]; its effects on breast cancer need to be further clarified. Flunarizine, a selective calcium entry blocker with calmodulin-binding properties and histamine H1-blocking activity has been reported to inhibit tumor cell growth in melanoma and lymphoma through inhibiting the Wnt pathway [24]. Flunarizine mediated cell autophagy by degrading N-Ras induction to inhibit growth of basal-like tumor cells *in vitro* and *in vivo*, with low toxicity; thus, it should be the subject of further investigation as a potential targeted therapy [25].

Second, upregulated DEGs mainly associated with endodermal cell differentiation and tumor behavior; generally, tumors with immature differentiation are much more aggressive than those with more mature differentiation. In breast cancer, stem cell activation and inhibition of cell differentiation are associated with tumorigenesis. Hypoxia is correlated with tumor differentiation, and increased protein levels of HIF-1 and HIF-2 are linked to worse prognosis of breast cancer patients [26]. Wnt/β-catenin pathway, a classic pathway in the formation of cancer stem cells, could enhance tumor growth through inhibiting cell differentiation [27]. Cell component GO enrichment analysis showed that the DEGs were mainly associated with the proteinaceous ECM. The ECM is an important factor in tumor growth, migration, and vascular formation in human breast cancer [28]. Cancer-associated fibroblasts have been found in the ECM and shown to have a role in breast cancer growth and chemoresistance. Hoang et al. found that ERK5, a member of the MAPK family, regulated the ECM to induce tumor proliferation and migration in triple-negative breast cancer [29]. Molecular function GO enrichment analysis indicated that the DEGs were mainly associated with lipid binding. Abnormal lipid metabolism is closely linked with tumorigenesis [30]. FAC, a key enzyme in fatty acid biosynthesis, acts as a metabolic oncogene in cancer growth. In breast cancer, HBXIP could modulate abnormal lipid metabolism and tumor growth by activating FAS signaling [31]. The long-chain ω-3 fatty acids have an important role in inflammation resolution, inhibiting breast cancer occurrence through production of lipid mediators. A meta-analysis of 16 cohort studies indicated that ω-3 intake was associated with a reduction in breast cancer risk [32]. The KEGG pathway analysis for DEGs mainly focused on ECM–receptor interactions, which have important roles in tumor growth and migration [33].

Third, four hub genes that could have important key functions in tumor growth in breast cancer, MMP1, SDC1, CD24, and SPP1, were identified using GEO and TCGA public datasets as potential prognostic biomarkers. In breast cancer tissue samples, MMP1, SDC1, CD24, and SPP1 showed higher protein expression compared with that in tumor-adjacent tissues, and their expression was positively correlation with tumor subtype and grade. MMP1, a member of the matrix metalloproteinase family, functions as an interstitial collagenase and fibroblast collagenase. Higher MMP1 expression could predict worse disease-free survival (DFS) and OS in patients with invasive breast cancer, but the mechanism underlying this association is not clear [34]. In the breast cancer xenograft model, reduction of MMP-1 expression significantly inhibited breast cancer growth, brain metastasis, and lung metastasis through reducing TGFa release and phosphor-EGFR expression [35]. In triple-negative breast cancer tissues, MMP1 protein expression positively depended on lymph node metastasis; furthermore, in an *in vitro* study, knockdown of MMP1 inhibited cell proliferation in triple-negative breast cancer MBA-231 cells [36]. In MCF-7 breast cancer cells, MMP1 is activated by Slug and enhances multidrug resistance; knockdown of Slug reduced MMP1 expression in these cells, further enhancing adriamycin resistance [37]; in immune analysis, in luminal breast cancer, MMP1 is negatively associated with tumor survival. Our results are almost consistent with those of previous studies. MMP1 has potential as a prognostic biomarker and therapeutic target in breast cancer, but more *in vivo* and clinical studies are required.

CD24 is a small and heavily glycosylated mucin-like glycosylphosphatidyl-inositol-linked cell surface protein, was detected in several types of carcinomas but is rarely expressed in normal tissues [38]. In breast cancer, the effects of CD24 on prognosis in terms of OS and DFS are controversial. Meta-analysis with breast cancer tissues indicated that higher CD24 expression was associated with shorter OS and correlated with tumor stage and lymph node metastasis [39]. Moreover, Jing et al. found that CD24 overexpressed in cancer tissues than in normal breast tissue commonly; in addition, CD24 expression was closely correlated with SDC1 mRNA expression, indicating that it could serve as a prognostic indicator for breast cancer [40]. In our study, CD24 protein expression was positively correlated with tumor grade, subtype and SDC1 expression, with higher expression of CD24 in breast cancer tissues compared with tumor-adjacent tissues. CD24 could thus be a prognostic biomarker and therapeutic target in breast cancer, but more *in vitro* and *in vivo* studies are needed to clarify its potential.

SDC1, a heparin cell surface proteoglycan that functions as a growth factors and chemokines co-receptor, which strongly correlated to the tumor aggressiveness and clinical results [41]. In breast cancer, higher expression of SDC1 is correlated to worse OS and positively correlated with grade. Cui et al. used the database to identify SDC1 as positively associated with PLAU expression and a potential prognostic marker and target in breast cancer [42]; however, the clinical effects and prognostic value were controversial. Knockdown of SDC1 could increase cell adhesion and motility dependent on integrin expression and IL6 secretion [43]. SDC1 is positively correlated with tumor subtype and grade according to our study and could thus become a prognostic biomarker. In basal breast cancer, SDC1, CD4+ T cells, and DCs were negatively correlated with tumor survival; however, the associated molecular mechanism needs to be further verified.

SPP1 is a protein overexpressed in breast tumors. Higher plasma levels of OPN with shorter OS in patients through inducing the tumor burden[44]. In our study, SPP1 expressed much higher in breast cancer tissue than in para cancerous tissues. SPP1 expression was correlated with the Luminal B breast cancer tissue.

Fourth, the higher expression of MMP1, CD24, SDC1 and SPP1 enriched in NK cell-mediated cytotoxicity and T cell receptor signaling pathway according to GSEA analysis indicated that immune cell infiltration might be correlated with expression of hub genes. High expression of MMP1 was positively correlated with infiltration of CD4+ T cells, CD8+ T cells, DC, neutrophils, and macrophages. In dermatofibrosarcoma protuberance tumor tissues, MMP1 was found to be prominent in tumor-associated macrophages [45]. CD24 expression was positively correlated with CD8+ T cell and neutrophil infiltration. In ovarian and triple-negative breast cancers, CD24 signaling could serve as a target for cancer immunotherapy through enhancing tumor-associated macrophage expression of Siglec-10 [46]. CD24 can aggravate acute liver injury via expression of IFN-γ on CD4+ T cells [47]. CD24 has potential as an immune therapy target in breast cancer, but further *in vitro* and *in vivo* studies are needed to clarify this. SDC1 expression was significantly positively correlated with infiltration of CD8+ T cells, macrophages, DC, and neutrophils. In pancreatic cancer, SDC1 regulates micropinocytosis to enhance tumor growth [48]. More studies are needed to explore whether SDC1 could be used as an immune therapy target in breast cancer. SPP1 was positively linked with infiltration of CD4+ T cells, CD8+ T cells, macrophages, neutrophils, and DC. In lung cancer, SPP1 enhanced PD-L1 expression and mediated macrophage polarization to facilitate immune escape [49]. SPP1 could have an important role as an immune therapy target in breast cancer.

In our results, high expression of MMP1, CD24, SDC1, and SPP1 correlated to the development of breast cancer, worse OS and immune cell infiltration, indicating that MMP1, CD24, SDC1, and SPP1 might be as the potential prognostic biomarkers and immunotherapy targets for breast tumor. We verified the results in several different datasets, including our own clinical datasets. However, there were some limitations of our study. First, there was a lack of *in vitro* and *in vivo* studies to verify the results, some other datasets were not used for verification the hub genes and immunity, like ArrayExpress database, InSilicoDB and METABRIC [50,51]. Second, further study of the roles of the four hub genes in different subtypes of breast cancer is required. Third, the survival results of our own clinical datasets not be analyzed. Finally, the molecular status of the four hub genes with respect to mutation and methylation was not checked in this study. Hence, further study is required to determine whether MMP1, CD24, SDC1, and SPP1 could be used as biomarkers or immune therapy targets in breast cancer.

## Supplementary Material

Supplementary Figures S1-S3 and Table S1

## Data Availability

The data that support the findings of the present study are available from the corresponding authors upon reasonable request.

## Abbreviations

CMap: Connectivity Map
DC: dendritic cell
DEG: differentially expressed gene
DFS: disease-free survival
ECM: extracellular matrix
EGA: European Genome-Phenome Archive
ER: estrogen receptor
FFPE: formalin-fixed, paraffin-embedded
GEO: Gene Expression Omnibus
GEPIA: Gene Expression Profiling Interactive Analysis
GO: Gene Ontology
GSEA: gene set enrichment analysis
HER2: human epidermal growth factor receptor-2
KEGG: Kyoto Encyclopedia of Genes and Genomes
MCODE: Molecular Complex Detection
OPN: osteopontin
OS: overall survival
PPI: protein–protein interaction
PR: progesterone receptor
STRING: Search Tool for the Retrieval of Interacting Genes
TCGA: The Cancer Genome Atlas
TMA: tissue microarray analysis
