# Supplementary material for: Identification of potential immunotherapy biomarkers for breast cancer by bioinformatics analysis
Source: Biosci Rep. 2022 Feb 4;42(2):BSR20212035. doi: 10.1042/BSR20212035 (PMC8819662; doi:10.1042/BSR20212035)
Supplement: Supplementary Figures S1-S3 and Table S1 [file BSR-2021-2035_supp.pdf]

Figure S1

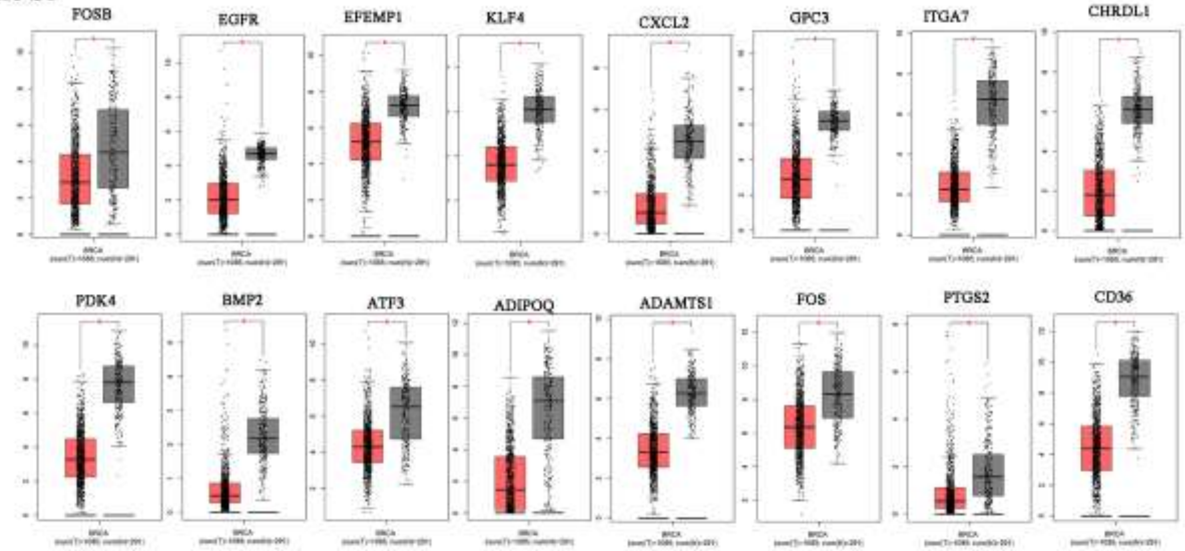

Figure S1: Expression levels of 17 hub genes were significantly downregulated in breast cancer tissues compared with normal breast tissues according to GEPIA.

Figure S2

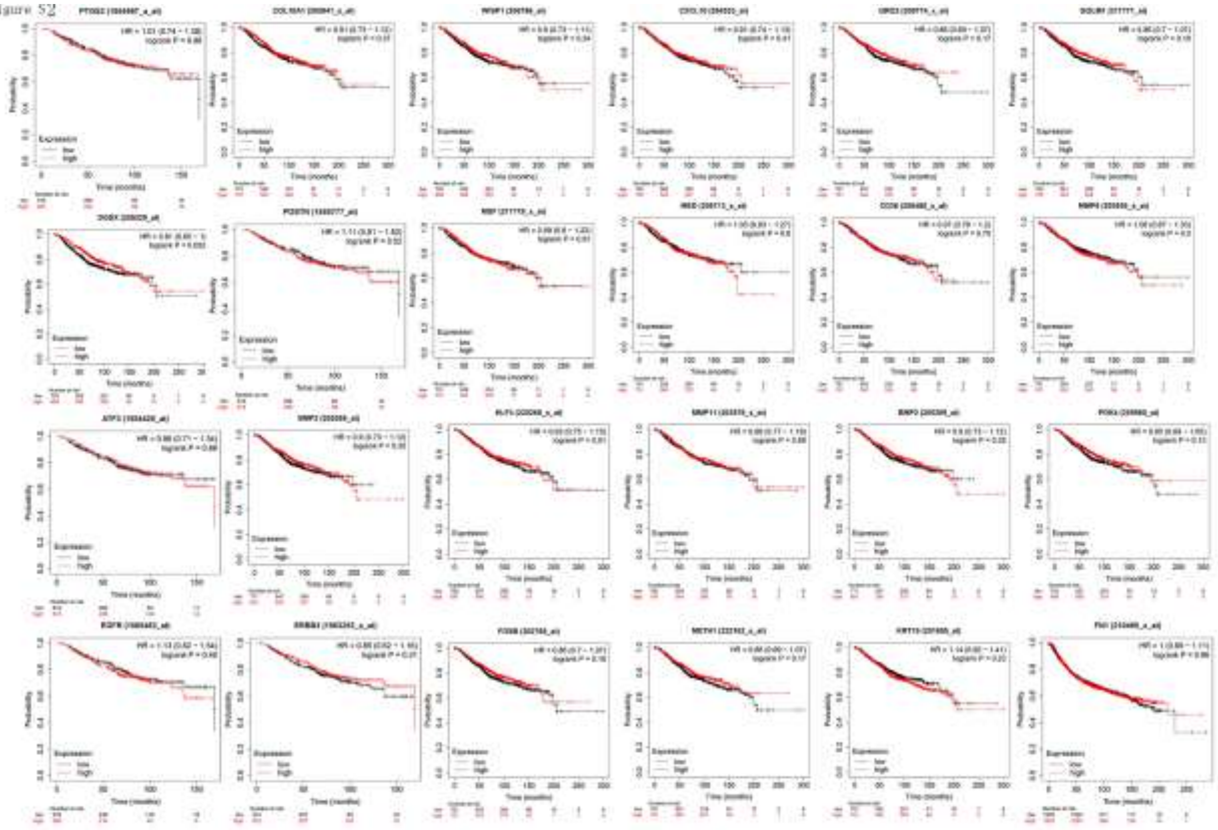

Figure S2: Expression levels of 24 hub genes were not correlated with OS in breast cancer patients.

Figure S3

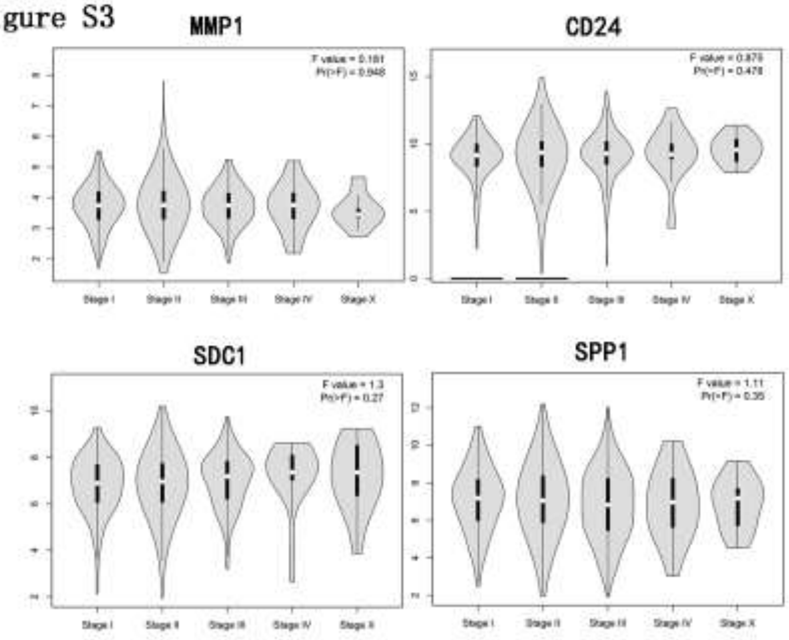

Figure S3: Violin plot showing expression of four key genes in different major pathological stages of breast cancer.

Table S1 All 121 commonly differentially expressed genes (DEGs) were detected from two profile datasets, including 90 downregulated genes and 31 up-regulated genes in the breast cancer tissues compared to normal breast tissues

DEGs	Genes Name
**Up-regulated**	VDR C15orf48 GOLM1 CD24 INHBA POSTN ERBB3 SDC1 MICAL2 GJB2 LOC101928269///LOC100506403///RUNX1 MMP9 MMP11 MIR8071-2///MIR8071-1///IGHV4-31///IGHM///IGHG2///IGHG1 ENC1 SHISA2 CXCL10 COMP FN1 SPP1 SULF1 KRT19 WISP1 BMPR1B COL10A1 LRRC15 COL11A1 TRIM59 FBXO32 MMP1 ISG15
**Down-regulated**	EMCN CHRDL1 GPIHBP1 ITIH5 ADAMTS1 SCARA5 BTNL9 IGS F10 SDPR EXOSC7///CLEC3B MEOX1 ATF3 CXCL2 ABCA6 EGFR LIFR LYVE1L ADIPOQ ITGA7 FOS LEPROT///LEPR BCHE PALMD MEOX2 CD36 FAM162B DCLK1 APOD E BF2 PIR-FIGF///FIGF GPR146 RASD1 SPRY2 CELF2 ITM2A HBB FHL1 KLF4 TFPI ABCA9 EMP1 PGM5-AS1 FMO2 PKD1L2 ADH1B ABCA8 TMEM132C SVEP1 IL33 SEMA3G TGFBR3 AKR1C 1 ANK2 ZBTB16 ASPA CCL15-CCL14///CCL14 FABP4 EDNRB GPC3 ACACB ADM TNMD DLC1 NOVA1 PTGS2 PGM5 GPX3 ADH1C FOSB EBF1 ENPP2 MAMDC2 TMTC1 PCOLCE2 LOC100506558///MATN2 CD300LG ABI3BP BMP2 TPPP3 RBP7 CCDC178 C2orf40 SFRP1 PDK4 COL6A6 CFD EFEMP1 P2RY14 TNXB///TNXA OC101930400///AKR1C2
